# Development of CPE/ssDNA-Based Electrochemical Sensor for the Detection of Leucine to Assess Soil Health

**DOI:** 10.3390/bios15110708

**Published:** 2025-10-22

**Authors:** Stella Girousi, Zoi Banti, Sophia Karastogianni, Rigini Papi, Dilsat Ozkan Ariksoysal, Evangelia E. Golia

**Affiliations:** 1 Laboratory of Analytical Chemistry, School of Chemistry, Aristotle University of Thessaloniki, 541 24 Thessaloniki, Greece; zbant@chem.auth.gr (Z.B.); skarasto@gmail.com (S.K.); 2 Laboratory of Biochemistry, School of Chemistry, Aristotle University of Thessaloniki, 541 24 Thessaloniki, Greece; rigini@chem.auth.gr; 3 Department of Analytical Chemistry, Faculty of Pharmacy, Ege University, 35100 Bornova, Izmir, Turkey; dilsat.ariksoysal@ege.edu.tr; 4 Laboratory of Soil Science, School of Agriculture, Aristotle University of Thessaloniki, 541 24 Thessaloniki, Greece; egolia@auth.gr

**Keywords:** leucine, square wave voltammetry, ssDNA, carbon paste electrode, electrochemical biosensor, soil

## Abstract

For the first time, the interaction between the amino acid leucine (Leu) and thermally denatured single-stranded (ss) DNA has been demonstrated by applying voltammetry. As a result of interaction, the characteristic peak of ssDNA, due to the oxidation of guanine residues, decreased upon interaction time. The interaction behavior between leucine and ssDNA was also studied with UV–vis spectrophotometry; the obtained results are in good agreement with voltammetric ones. The results of the interaction study were exploited in order to develop a SWV method for the determination of leucine at the ssDNA-modified carbon paste electrode (CPE). Different parameters were tested to optimize the conditions of the determination. The peak of guanine was at around +0.86 V. Linearity was observed in the range of 0.213–4.761 μg/L (r = 0.9990) while LOD equals 0.071 μg/L. The method was applied to a spiked soil sample and gave satisfactory results.

## 1. Introduction

Amino acids are essential as they can support plants under extreme conditions, including high concentrations of potentially toxic elements, as well as extremely high or low temperatures [1,2] or high concentrations of salts and severe water shortages which may occur in the soil environment [3,4,5]. Leucine also has the potential to reduce oxidative damage by regulating a plant’s antioxidant system, according to a study on *Panax notoginseng* [6]. In the investigation of Bayranvand et al. [7] it was found out that leucine resulted in an increase in the activity of appropriate enzymes activating the nitrogen cycle in soils. Also, it can enhance the nitrogen metabolism [8] and subsequently it can lead to an increase in plant resistance to stress from high Cu concentrations [5].

Leucine is also used as an indicator of the stress that a plant organism undergoes when stressed by extreme toxicity conditions, as in the case of emerging pollutants and environmental hazards or where an amendment has been used in the soil tested [9].

Variations in the amount of soil organic matter can provide valuable information on possible scenarios of land use, fertility levels, the percentage of the land area under cultivation, as well as the cultivation method [10]. Agricultural soils usually contain higher amounts of organic matter, as they either contain manure or other organic soil amendments [11]. In urban soils, the organic matter is usually oxidized, and the amounts are low [12].

Soil plays an important role in reducing carbon footprint by storing and sequestering carbon dioxide from the atmosphere. A healthy and fertile soil can increase its carbon storage capacity, helping to reduce emissions [13]. Therefore, the existence of leucine could also lead to conclusions regarding soil health.

Clunes and Pinochet [14] in their research concluded that leucine presence in soils and its variation level provides a satisfactory assessment of the soil’s ability to retain soluble carbon compounds which are easily degradable. Furthermore, it provides an indirect estimation regarding the effects of the dynamics of land use changes on carbon sequestration capacity, and the way such changes are relevant to the biodiversity of soil micro-organisms [15]. In other words, it can be an indicator about the changes that climate crisis may induce in soils, affecting the microbiota and the rate of carbon sequestration [16].

Due to these important properties of leucine, research on its determination in both soil and plants is valuable. The extractant used depends on the soil fraction and the type of plant in which the concentration of leucine should be determined.

Leucine, like many other amino acids, can be isolated in the exchangeable soil fraction and in clay minerals, as it can be bound to them [14]. In such applications, leucine is determined in the aqueous extract of CH_3_COONH_4_ 0.1 M (pH = 7). More often, experiments involve the addition of quantities of the amino acid, followed by incubation for a specific period of time and under specific conditions, and then determination of the remaining amount of amino acid.

The method commonly used is based on the colorimetric determination after the ninhydrin reaction at pH of 5.0 [17]. However, this method’s main problem is that the soil extracts must be adjusted to pH = 5 and this may modulate significant parameters in the remaining components of the soil extract, resulting in fluctuations in the amount of amino acid that is actually free (rather than bound) in order to be quantified [14].

Therefore, a method that could be carried out at pH values ranged between 5 and 8 would be preferable.

To quantitatively determine leucine in soil samples, the researchers developed an HPLC method combined with mass spectrometry [18], utilizing 6-aminoquinolyl-N-hydroxysuccinyl imidyl carbamate (AQC). Linearity was noted within the range of 50–800 mol L^−1^.

Detection limits were 0.20–0.60 pmol μL^−1^ in the column and 0.07–0.24 μg g^−1^ in soil.

Amino acids, in free or polymeric form, contribute significantly to most ecosystems’ nitrogen levels, and so play an important role in the soil nitrogen cycle. Some plants’ ability to absorb amino acids straight from the soil could offer a competitive edge, especially in environments with limited nitrogen availability. Inorganic nitrogen levels (NH_4_ and NO_3_^−^) are frequently assessed in soil solutions or soil water/KCl extracts, but an additional method is needed to evaluate free amino acids and all accessible plant pools.

The purpose of this research [14] is to create and evaluate a procedure for rapidly and sensitively determining total free amino acids in soil solutions and soil extracts (water or 2 M KCl). The spectrofluorometric technique is based on the reaction of free amino acids with o-phthaldialdehyde and b-mercaptoethanol. The fluorometric method is substantially more sensitive (working range 0.1–50 mM) than standard spectrophotometric analysis procedures for free amino acids, which use the ninhydrin reagent (10–500 mM). Furthermore, the method requires only tiny sample quantities, is quick and easy to use, and has linearity in the range 0.1–100 mM.

Free amino acid concentrations were measured in a variety of environments, including highland and lowland grasslands, woods, heathlands, and coastal saltmarsh. Overall, the concentration of free amino acids in soil solution was low and did not vary by soil type. Free amino acids typically account for 10–40% of total soluble N in soil solutions, constituting a significant soluble N and plant-accessible pool in soil.

In recent decades, extensive research in electrochemistry has advanced the detection of biological molecules. Among these, amino acids (AAs) have garnered significant interest from scientists and researchers. Electrochemical sensors and biosensors are simple to use, while presenting high selectivity, sensitivity, and time-saving abilities.

Regarding analytical methods for the determination of leucine, an important comparison of analytical methodologies is being discussed in [19].

In the mentioned work [19], two main categories of analytical techniques are being discussed. On the one hand, separation techniques, like thin layer chromatography, capillary electrophoresis, gas chromatography, and high pressure liquid chromatography, which are quite old, at around 25–50 years old, are time-consuming (20–40 min/ sample) and require specialized equipment. On the other hand, optical techniques lie, like spectrophotometric ninhydrin method and the fluorometric detection of amino acids, a technique that relies upon the reaction of amino acids with o-phthaldialdehyde and b-mercaptoethanol (OPAME). The proposed analytical methods, so far in the literature, determine leucine at a concentration level of 10^−3^ mol/L. A more recent publication [20] employing capillary electrophoresis with laser-induced fluorescence achieved measurement of leucine in a soil sample at a 10^−6^ mol/L concentration level, while hyphenated analytical techniques gave an LOD of 13–384 ng·g^−1^ (dry soil basis) and an LOQ of 43–1267 ng·g^−1^ (dry soil) [21], while in [22] they gave a level of µmol/g dry sample.

In the present work, it is possible to determine leucine at a concentration level of 10^−8^ mol/L in soil sample by employing an easy-to-build electrochemical sensor.

A comparison of the analytical characteristics of the proposed method with the already established ones so far in the literature, mainly in biological substrates, and with the absence of applications in soil samples underlined, is presented in Table 1.

DNA immobilization at electrode surfaces has been identified as a promising technique for producing a conductive thin layer with nanostructures, hence enhancing the electrode’s surface area for the future fabrication of efficient biosensors [29]. Furthermore, this approach can create thin coatings with negative charges on the electrode surface, allowing for the adsorption of positively charged target molecules while reducing unwanted adsorption on the substrate. Typically, DNA is coated on the surface of conductive materials to create a bilayer modified electrode. The use of conductive materials on an electrode can increase its surface area and interfacial conductivity. These bilayer modified electrodes are capable of sensitively detecting tiny compounds, including medicines, carcinogens, and pollutants, that interact with DNA.

A rare metabolic disease caused by large amounts of branched-chain amino acids (b AAs), i.e., leucine, isoleucine, and valine, and reported MSUD and b Aas, was studied as an assay based on electrochemical (bio)sensing [29].

The interaction study between leucine and DNA can be the main trend in the detection of amino acids with electrochemical (bio)-sensors in the use of biomolecules. In general, all electrochemical approaches in both simple electrodes and advanced biosensors may be suitable for the electrochemical detection of amino acids, due to the low detection limit required.

However, simple electrodes are probably not the most suitable solution in the analysis of complicated samples, since biomolecules improve the selectivity, sensitivity, and reproducibility of the analysis. In this sense, the damage resistance of biomolecule modified electrodes is particularly important, since they can perform various analyses without altering their analytical characteristics.

In the literature, DNA interaction studies along with leucine were realized in the following studies, in which it is being concluded that the aliphatic amino acids alanine, isoleucine, leucine, and valine show low propensity in both binding specificity groups [30,31,32], while leucine interaction with DNA is due to the shortness of its side chain [30].

Another review discusses the structure and function of single-stranded DNA (ssDNA) binding proteins (SSBs), as well as the structural characteristics that determine SSB binding selectivity. Machine learning-based approaches to predicting SSBs from double-stranded DNA (dsDNA) binding proteins (DSBs) are extensively studied [31].

All types of amino acid side chains interact more favorably with ssDNA, with the exception of positively charged side chains, and aromatic and aliphatic side chains intercalate particularly well. In ssDNA, positively charged side chains and sodium ions bind to cytosine, whereas negatively charged side chains and chloride ions attach to guanine. The work demonstrates the intercalation of a leucine side chain between bases [32].

The aliphatic amino acids alanine (A), isoleucine (I), leucine (L), and valine (V), as well as the negatively charged glutamate (E) and cysteine (C), have a low affinity for ssDNA in both the selective binding (SP) and nonspecific binding (NS) groups [33].

The conclusions of the above-mentioned studies showed that important findings that are in favor of ssDNA-leucine interactions exist.

Considering the foregoing, the presence of high leucine levels may be an indicator of urban or rural origin of the soil samples. High concentrations of leucine indicate agricultural soil, and low concentrations indicate urban soil and less fertile soil. Therefore, the analytical method we propose could help a traceability tool of the soil samples analyzed to be a measure of fertility and/or urban or agricultural origin.

In this context, the objective of the proposed study is the development of an electrochemical ssDNA biosensor for the detection of leucine. For the first time, the interaction between the amino acid leucine (Leu) and thermally denatured single-stranded (ss) DNA has been demonstrated by applying voltammetry. As a result of the interaction, the characteristic peak of ssDNA, due to the oxidation of guanine residues, was exploited for the development of a promising sensor. In particular, in this study, the methodology of preparation, their voltammetric behavior, the conditions of DNA immobilization, and the analytical characteristics of the sensor were studied.

## 2. Materials and Methods

### 2.1. Chemicals and Reagents for the Development of DNA Biosensor

Double-stranded deoxyribonucleic acid (dsDNA) from fish sperm was supplied from Sigma-Aldrich (Darmstadt, Germany) as a lyophilized powder. Single-stranded deoxyribonucleic acid (ssDNA) was prepared by thermal denaturation. dsDNA (1000 mg L^−1^) was heated in 10 mM Tris-HCl (8) at 100 °C for 15 min and was immediately placed in iced bath for cooling for 20 min. The stock solution of DNA gave a ratio of UV absorbance at 260/280 nm of ~1.90 absorbance, indicating that the DNA was sufficiently free of protein contamination. In this work, ultra-pure water (18 Ω) was used for the preparation of all solutions (Elgastat, Buckinghamshire, UK), and the chemicals used were of analytical grade. Experiments were performed at room temperature (22.0–25.0 °C).

### 2.2. Solutions

Solvents, acids, bases, and standard solutions were all analytical grade. They were used as received unless otherwise noted. Sodium hydroxide (NaOH), acetic acid (CH_3_COOH), potassium dihydrogen phosphate (KH_2_PO_4_), tris hydroxymethyl amino-methane (Tris 99.8%, ACS), and hydrogen chloride (HCl) were supplied from Merck (Darmstadt, Germany). Potassium chloride (KCl), potassium iodide (KI), potassium fluoride (KF), sodium chloride (NaCl), and sodium fluoride (NaF) were supplied from Merck (Darmstadt, Germany). Amino acids L-leucine (Leu), L-isoleucine (Ile), L-valine (Val), and L-methionine (Met) CELLPURE^®^ ≥ 99% were purchased from Carl ROTH GmbH + Co. KG (Karlsruhe, Germany), while L-phenylalanine (Phe) ≥ 98% was from Tokyo Chemical Industry Co., Ltd. (TCI, Kyoto, Japan). All aqueous solutions were prepared with deionized water. A magnetic stirring bar of 8 × 3 mm, PTFE (HEINZ HERENZ HAMBURG, Hamburg, Germany), was also used.

### 2.3. Apparatus

The voltammetric analysis was conducted using a PalmSens potentiostat/galvanostat (PalmSens, Houten, Netherlands), The Netherlands. The electrochemical cell employed in the experiment consisted of CPE with a 3 mm inner and 9 mm outer diameter for the PTFE sleeve which was used as a “working electrode”, an Ag/AgCl reference electrode (RE) saturated with 3 mol·L−1 KCl, and a platinum wire counter electrode (CE) (Metrohm, Herisau, Switzerland). All weighings were performed using Sartorius-type scales (Kernew 220-30014 and Denver Instrument XE-310, East Lyme, CT, USA), with procedures conducted at ambient temperature and solution pH measured using a Consort C830 pH meter. The electrochemical cells (with a 25 mm diameter) were washed and rinsed with deionized water and cleaned with dilute nitric acid.

### 2.4. UV–Vis Study of ssDNA Along with Leucine

Absorption titrations were obtained on a Shimadzu 160A spectrophotometer (Shimadzu, Tokyo, Japan) by using a fixed leucine concentration (75 mM) to which increments of the ssDNA stock solution were added. Leu–DNA solutions were allowed to incubate for 15 min before the absorption spectra were recorded with the UV region from 200 to 450 nm. Absorption titrations were carried out by employing the Wolfe–Shimmer equation [34]: [DNA]/(|ɛA − ɛF|) = [DNA]/(|ɛB − ɛF|) + 1/{Kb (|ɛB − ɛF|)}, where [DNA] is the concentration of DNA in base pairs; ɛA, ɛF, and ɛB correspond to Absd/[compound], the extinction coefficient of the free leucine, and the extinction coefficient of the compound in the fully bound form, respectively. In the plot of [DNA]/(|ɛA − ɛF|) versus [DNA], the intrinsic binding constant (Kb, in M^−1^) is then given by the ratio of the slope to the intercept.

## 3. Procedures

### Interaction of ssDNA with Leu at the CPE/ssDNA-Based Biosensor with Leucine

The carbon paste was prepared in the usual way by hand-mixing graphite powder and nujol oil. The ratio of graphite powder to nujol oil was 75:25. The resulting paste was packed tightly into a Teflon sleeve. Electrical contact was established with a stainless steel screw. The surface was polished to a smooth finish before use.

CPE was preconditioned by applying a potential at +1.7 V for 1 min without stirring. The modification procedure involves the following:

(a) ssDNA immobilization at the CPE surface; CPE was modified with 100 μL from a 190 mg L^−1^ ssDNA standard solution (concentration in the electrochemical cell 3.8 mg L^−1^ ssDNA), in a stirred sample solution (0.2 M acetate buffer solution pH 5.0) for 60 s at +0.5 V.

(b) Interaction of Leu with CPE/ssDNA-based biosensor; ssDNA-modified CPE allowed to interact, with stirring at open circuit with leucine for 60 s in Tris-HCl 0.1 M + 0.008 M NaCl, pH = 8 solution.

(c) The transduction was performed in 0.1 M acetate buffer solution pH 5.0.

**Scheme 1:**
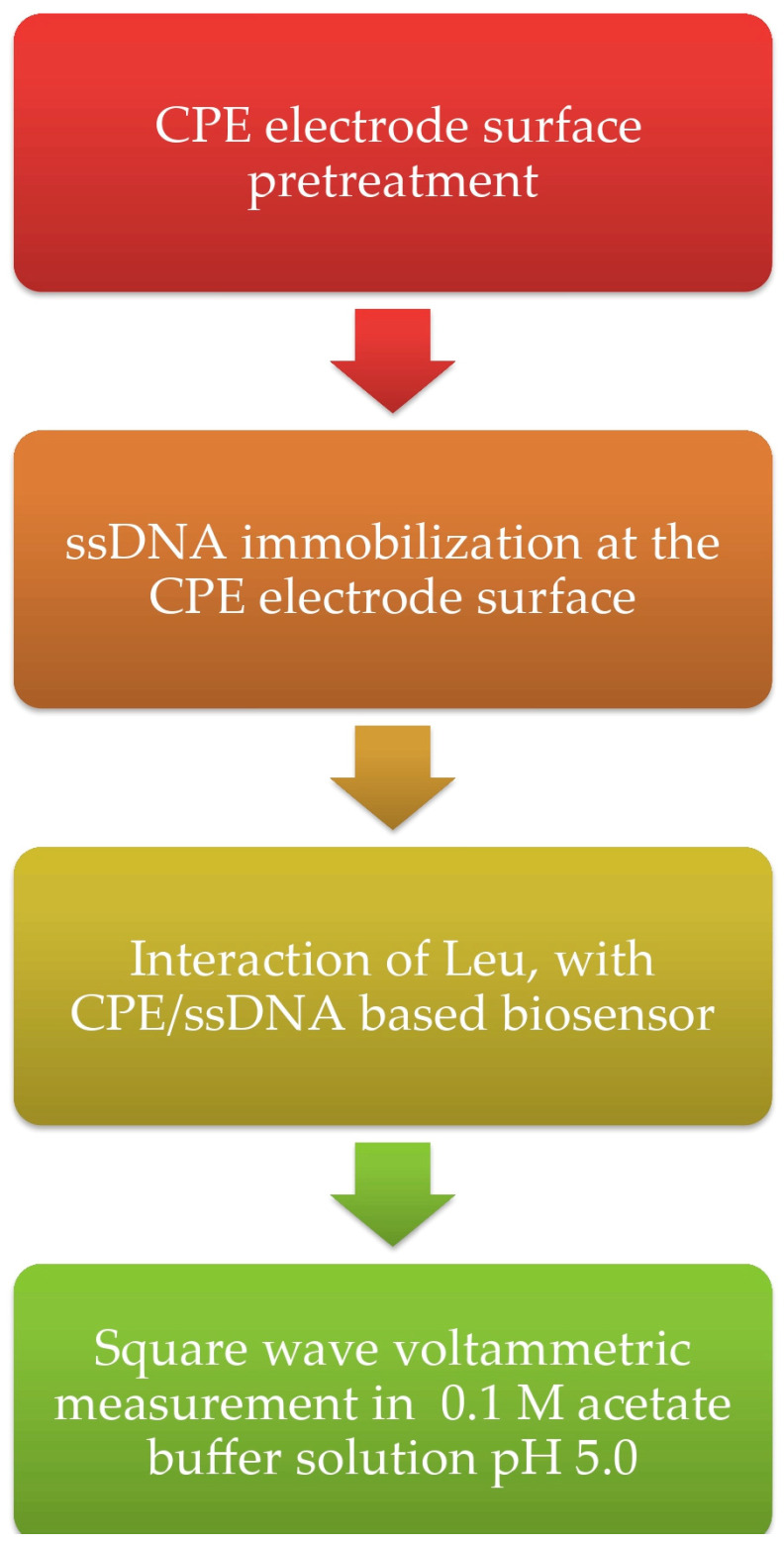
Experimental procedure.

The procedure is summarized in Scheme 1.

Before each medium swap, the electrode was properly washed with water for 5 s. In all processes, transduction was carried out in a blank acetate buffer solution, and square wave voltammetry (SWV) was used under the following conditions: Estep = 0.005 V, Epulse = 0.015 V, and frequency = 12 Hz (unless otherwise noted). The studied potential ranged from 0 to 1.3 V.

Native ssDNA yielded in this medium two positive peaks, the first within 0.82 and 0.86 V where guanine [G] residues are being oxidized, and the second between 1.05 and 1.15 V was attributed to adenine [A] residues.

It must be noted that three experimental replicates were performed in all of the subsequent studies, unless stated otherwise.

## 4. Results and Discussion

In order to favor the performance of the biosensor, various factors influencing the response of the biosensor, such as ssDNA concentration, concentration of salts, applied potential, and accumulation time, were investigated.

The current height of the oxidation peak of guanine and adenine residues increases with increasing ssDNA concentration up to 3.8 mg L^−1^. Above this value, saturation of the CPE surface is observed, and the current stabilizes. Thus, the concentration of 3.8 mg L^−1^ is chosen as the most appropriate for the following studies, since the electrode coverage is complete.

The influence of different salts was studied, like NaCl, NaF, KI, KF, and KCl. The effect of ionic strength was also studied as a parameter influencing the electrochemical behavior of ssDNA, and a concentration of 0.005 M NaCl was found to be ideal.

**Figure 1:**
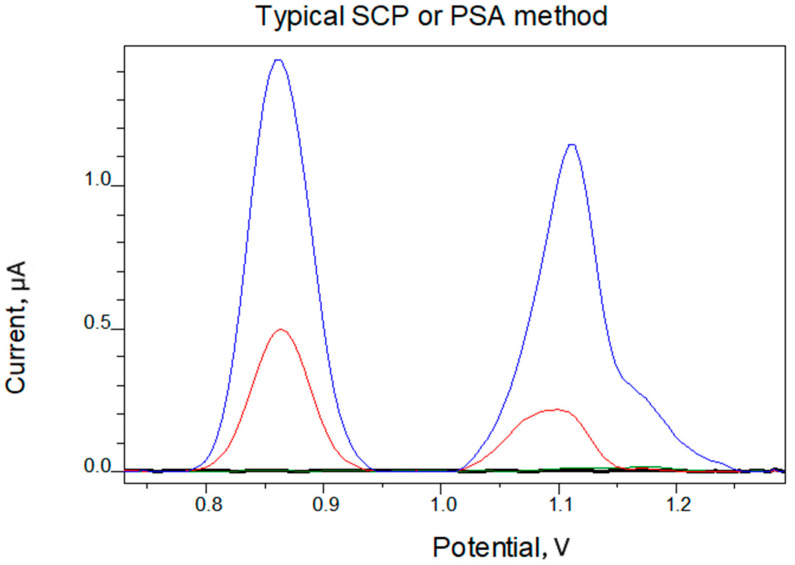
Bare CPE (black line), bare CPE +leucine (green line), CPE/ssDNA (blue line), CPE/ssDNA +leucine (red line). Conditions: 3.8 mg L^−1^ ssDNA, 1.15 μg L^−1^ Leu, interaction time 60 s, and other experimental and instrumental conditions described. Acetate buffer solution (SWV): Estep = 0.005 V, Epulse = 0.015 V, and frequency = 12 Hz. Potential range 0–1.3 V.

Figure 1 shows the oxidation square wave voltammograms (SWVs) of bare CPE (black line), leucine on bare CPE (green line), ssDNA/CPE (blue line), and ssDNA/CPE after its interaction with leucine (red line). The interaction with leucine reduced the oxidation peak current on the CPE/ssDNA at +0.850 V (guanine) and +1.100 V (adenine), resulting in a minor shift in the oxidation peak potential to more negative values of roughly +5 mV and +15 mV. Furthermore, no oxidation peaks for leucine were detected on the bare CPE. This event, combined with the shift in the peak potential of ssDNA residues, suggests that leucine may interact with the constructed electrode ssDNA/CPE.

**Figure 2:**
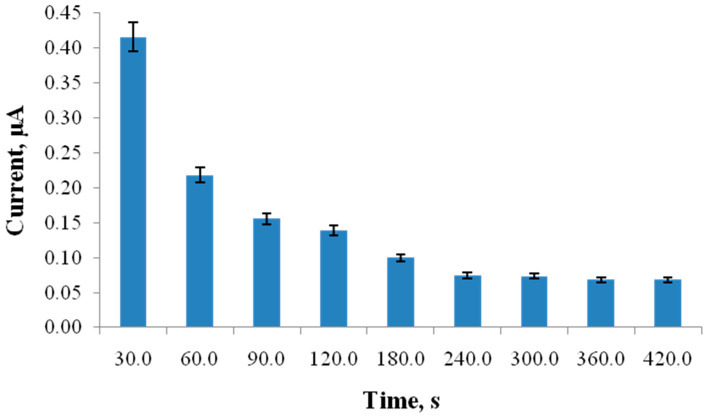
Dependence of the oxidation of guanine peak current upon interaction time with constant concentrion of leucine, Leu. Experimental conditions: 3.8 mg L^−1^ ssDNA, 1.15 μg L^−1^ Leu and under the optimum experimental conditions; Acetate buffer solution (SWV): Estep = 0.005 V, Epulse = 0.015 V, and frequency = 12 Hz. Potential range 0–1.3 V.

The interaction time has a profound effect on the SWV response. The selection of the interaction time depended on changes in the characteristic oxidation peak of guanine in ssDNA. Increasing the interaction time of leucine with ssDNA, which is a relaxed and more accessible form of DNA, alters the current height of the characteristic oxidation peak of guanine and adenine residues in DNA. These alterations are directly tied to steric changes in the DNA backbone, as well as its capacity to attach to the carbon paste’s hard surface. Figure 2 depicts how the oxidation of guanine and adenine peak current varies with interaction time with Leu. It is demonstrated that the current response decreases as the interaction time increases. The observed decrease in the peak current intensity of oxidation of guanine residues during the studied time period may be due either to some kind of equilibrium between the free and ssDNA-bound complex, or to steric changes in the ssDNA structure which lead the electroactive groups of ssDNA to unfavorable positions for oxidation on the electrode surface, a fact that means that the binding reaction of leucine with ssDNA reaches equilibrium. Consequently, optimum interaction time was found to be 60 s and was chosen for subsequent experiments. It should be noted that the adenine oxidation peak is not reproducible, and, for this reason, only guanine residues were selected for study. Conclusively, this behavior cannot be attributed to electrostatic interaction phenomena as leucine is a neutral molecule; it is the result of the binding of Leu to the immobilized ssDNA which resulted in the decrease in both characteristic oxidation peaks of ssDNA [35,36,37,38,39].

**Figure 3:**
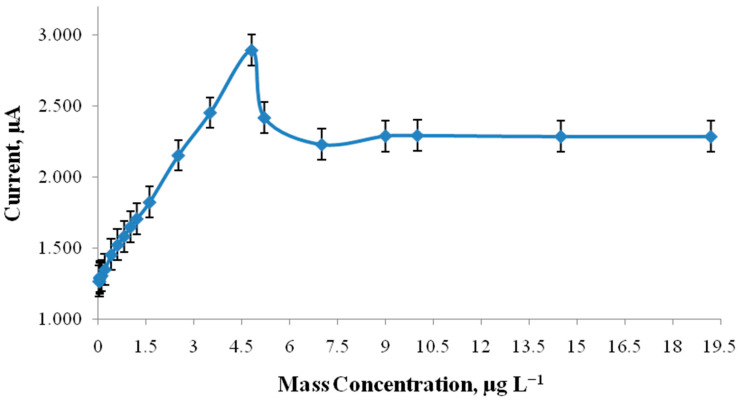
Dependence of the characteristic oxidation peak of guanine with increasing concentrations of leucine and constant interaction time. Experimental conditions: 3.8 mg L^−1^ ssDNA, 60 s interaction time and under the optimum experimental conditions.

Figure 3 shows the dependence of the characteristic oxidation peak current of guanine on increasing concentrations of Leu, under the optimal experimental conditions at a given interaction time of 60 s. Up to the concentration 4.761 μg L^−1^, a subsequent increase in the ssDNA peak was observed. Above that value, it falls up to 7.000 μg L^−1^, then reaches a plateau, indicating that the reaction between Leu and DNA has reached equilibrium. In the presence of Leu, no new redox peaks form, and the peak potential remains constant. This type of behavior shows that the interaction between Leu and ssDNA is mass concentration dependent. When Leu interacts with immobilized ssDNA at the CPE, the molecule binds preferentially to the biomolecule’s solution-facing sites. We must consider that ssDNA is electrostatically adsorbed at the CPE surface via the negatively charged sugar-phosphate backbone, resulting in a flat layer on the electrode surface. Furthermore, the side of ssDNA that comes into direct touch with the electrode surface is difficult to access. At low concentrations of Leu, the peak of ssDNA is increased because either of the molecules of Leu are bound to the side of the ssDNA opposite to its contact with the electrode surface. Therefore, at low Leu concentrations, an increase in the guanine signal is observed due to the electron transfer-enhancing effect of leucine on the guanine oxidation peak, rather than the DNA–Leu interaction [40].

The decrease in guanine oxidation signal at high mass concentration of Leu could be due to the bending of the ssDNA molecule and its ability to stick to the rough CPE surface. Furthermore, it can be explained in terms of the equilibrium mixture of the free and ssDNA-bound complexes at the electrode surface, or in terms of conformational changes in ssDNA structure that result in the steric placement of electroactive ssDNA residues on the electrode surface. In other words, because leucine binds to/interacts with the guanines in ssDNA, it partially protects them from oxidation. This results in a decreased signal. This could also be explained by a DNA–leucine interaction. Nonetheless, the data are insufficiently substantial to provide a safe conclusion.

Additionally, UV–vis absorption spectroscopy was utilized to study the in vitro interaction of leucine with ssDNA. This technique enabled the detection of variations in the electronic environment of ssDNA upon leucine addition by monitoring changes in the absorbance around 260 nm, the characteristic maximum wavelength of ssDNA. The Wolfe–Shimer equation was used to calculate the DNA-binding constant (Kb), which is the ratio of the slope to the y intercept in plots of [DNA]/(εA − εf) vs. [DNA]. The Kb value was determined to be 0.0326 M^−1^, indicating that leucine does not bind to DNA through intercalation, as expected since ssDNA was used. This binding affinity also implies that leucine may interact with ssDNA through transient hydrogen bonds or van der Waals interactions, instead of through insertion into the DNA backbone The obtained results of this study between ssDNA and leucine [41] are in good agreement with the performed voltammetric interaction study and the results presented so far in the literature [32,33,41].

**Figure 4:**
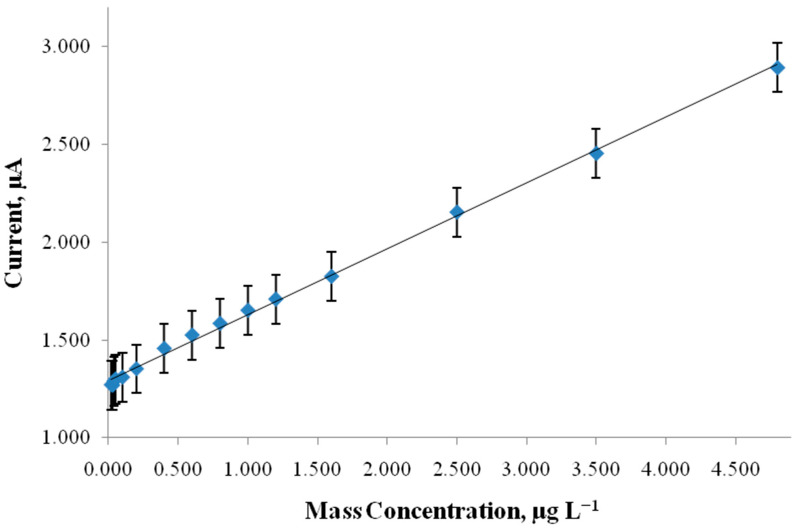
Calibration curve of Leu, under optimal experimental conditions (guanine oxidation signal is being measured). Acetate buffer solution (SWV): Estep = 0.005 V, Epulse = 0.015 V, and frequency = 12 Hz. Potential range 0–1.3 V.

After optimizing the biosensor’s main parameters, a calibration curve was plotted (Figure 4). The results demonstrated that linearity was observed in the concentration range of 0.213–4.761 μg/L, while LOD equals 0.073 μg/L (in standard solutions) and the regression equation: y = 0.3371 (±0.0042)x + 1.2913 (±0.0075) (*R*^2^ = 0.9980). It must be noted that the detection limit was determined by 3 *s*_b_/slope where *s*_b_ is the standard deviation of the blank measurements and the slope of the calibration curve.

### Interferences Study

Other amino acids may interfere with the detection of leucine. Amino acids, such as isoleucine, valine, phenylalanine, and methionine, were investigated as potential interferents. Thus, the influence of foreign compounds on the current responsiveness of the modified electrode was examined, as shown in Table 2. The interfering effect of the studied amino acids was evaluated related to recovery, R%.

## 5. Application in Soil Sample

The analyzed soil sample was a typical clayey loam soil of a rural area in the Greek countryside. For the extraction of leucine, shaking was performed for 1 h with an aqueous solution of CH_3_COONH_4_ 0.1 M (pH = 7), in a ratio of 1:10 (5 g of air-dried soil with 50 mL of solution). Subsequently, filtration was performed and the filtrate was subjected to 1:100 dilution with d.H_2_O (sample solution).

A CPE/ssDNA-based biosensor was transferred to the diluted and stirred sample solution and allowed to interact with leucine for 60 s in Tris-HCl 0.1 M + 0.008 M NaCl, pH = 8 solution. The transduction was performed in 0.1 M acetate buffer solution pH 5.0. A recovery of 98.7% was obtained by the proposed method using the standard addition method.

**Figure 5:**
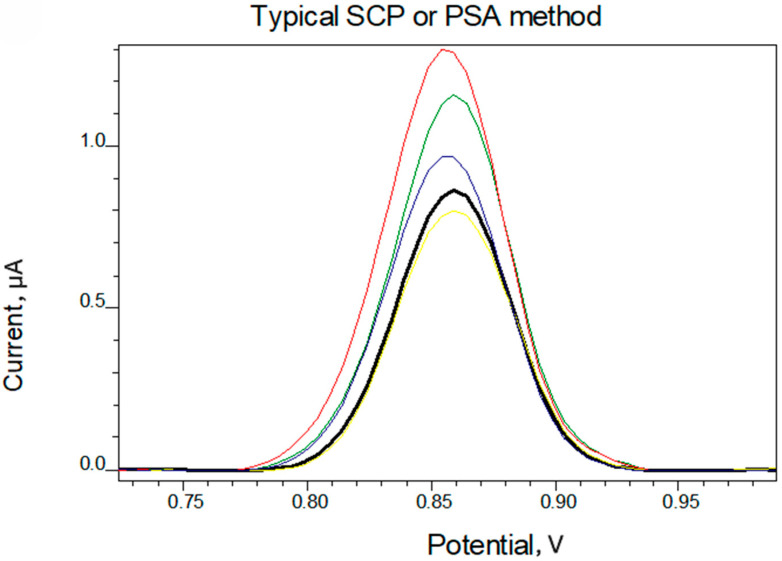
Overlay of standard additions in the soil sample extract under the optimum experimental conditions [yellow line, sample; black line, 1st standard addition (0.5 μg L^−1^); blue line, 2nd standard addition (1.0 μg L^−1^); green line, 3rd standard addition (1.5 μg L^−1^); red line, 4th standard addition (2.0 μg L^−1^)].

The corresponding voltammogram is presented in Figure 5. The regression equation for the soil sample was calculated to be y = 0.036x + 0.721 (R^2^ = 0.9994).

## 6. Conclusions

The current study is novel since it is the first effort to measure leucine utilizing square wave voltammetry (SWV) with a CPE/ssDNA biosensor. Finally, the suggested electrochemical sensor was shown to be very sensitive, with a lower detection limit than other techniques published in the literature, applied in samples of biological importance so far (serum, urine). The proposed biosensor is being developed based upon ssDNA’s interaction with leucine. Furthermore, the proposed approach was successfully applied to a spiked soil sample, demonstrating the sensor’s potential relevance to real-world sample analysis. The proposed method can cover a range of concentrations, that can meet requirements for leucine determination in both organic and nitrogen-rich soils (e.g., organic and/or clayey), as well as sandy, acidic, or saline soils, where low levels of leucine are expected [42,43]. Additionally, the development of a simple, cost-effective, and environmentally friendly ssDNA biosensor is being demonstrated, successfully applied in the selective and sensitive detection of leucine in standard solutions and real samples.

## Figures and Tables

**Table 1:** Comparison of the analytical characteristics of the already established methods with the proposed work.

Ref.	Application	LOD mol/L	Linearity mol/L	Electrode
[23]	blood	3 × 10^−12^	10^−11^–10^−8^	α-CD/ZnO/nanoC
[24]		8 × 10^−16^	25–700 × 10^−6^	v-NiNWs
[25]	spiked urine, milk and serum	37.5 ± 0.2 × 10^−12^	0.1–100 × 10^−9^	SrO NR
[26]	-			Diamond paste
[27]	-	2 × 10^−6^	10–600 × 10^−6^	Amperometric bienzyme ScPE
[28]	blood, urine samples	3.0 × 10^−6^	9.0 × 10^−6^–5 × 10^−3^	MWNTs
This work	spiked soil	4.9 × 10^−10^	1.4 × 10^−9^–3.5 × 10^−8^	CPE/ssDNA

**Table 2:** Interference study mass ratio 100:1.

Interfering Amino Acid	Recovery, R% at 3 μg L^−1^
Leucine	100
Isoleucine	108.75
Valine	100.95
Phenylalanine	102.4
Methionine	97.63

## Data Availability

The original contributions presented in this study are included in the article. Further inquiries can be directed to the corresponding author.
